# Outcomes and implementation challenges of using daily treatment regimens with an innovative adherence support tool among HIV-infected tuberculosis patients in Karnataka, India: a mixed-methods study

**DOI:** 10.1080/16549716.2019.1568826

**Published:** 2019-02-04

**Authors:** Pruthu Thekkur, Ajay MV Kumar, Palanivel Chinnakali, Sriram Selvaraju, Ramachandra Bairy, Akash Ranjan Singh, Abhay Nirgude, Kalaiselvi Selvaraj, Vinayagamoorthy Venugopal, Suresh Shastri

**Affiliations:** a Centre for Operational Research, International Union Against Tuberculosis and Lung Disease, New Delhi, India; b Centre for Operational Research, International Union Against Tuberculosis and Lung Disease, Paris, France; c Department of Preventive and Social Medicine, Jawaharlal Institute of Postgraduate Medical Education and Research (JIPMER), Puducherry, India; d Epidemiology Unit, National Institute for Research in Tuberculosis, Chennai, India; e Department of Health and Family Welfare Services, Bengaluru, Karnataka, India; f Department of Community Medicine, All India Institute of Medical Sciences, Bhopal, Madhya Pradesh, India; g Department of Community Medicine, Yenepoya Medical College, Mangalore, Karnataka, India; h Department of Community Medicine, Pondicherry Institute of Medical Sciences, Pondicherry, India; i Department of Community Medicine, Sri Manakula Vinayagar Medical College and Hospital, Pondicherry, India

**Keywords:** Self-administered treatment, m-health, e-health, 99DOTS, SORT IT, operational research

## Abstract

**Background**: In India, a new care package consisting of (i) daily regimen with fixed-dose combination drugs, collected once-a-month and self-administered by the patient, (ii) ‘one stop service’ at antiretroviral treatment (ART) centre for both HIV and tuberculosis (TB) treatment and (iii) technology-enabled adherence support (99DOTS, which required patients to give a missed phone call after consuming drugs) was piloted for treatment of TB among HIV-infected TB patients. Conventional care included intermittent regimen (drugs consumed thrice-weekly) delivered under direct observation of treatment supporter and the patients needing to visit TB and HIV care facilities, separately for treatment.

**Objective**: To assess the effect of new care package on TB treatment outcomes among HIV-TB patients registered during January–December 2016, as compared to conventional care and explore the implementation challenges.

**Methods**: A mixed-methods study was conducted in four districts of Karnataka, India where new care package was piloted in few ART centres while the rest provided conventional care. Quantitative component involved a secondary cohort analysis of routine programme data. Adjusted relative risk(aRR) was calculated using Poisson regression to measure association between new care package and unsuccessful treatment outcome. We conducted in-depth interviews with healthcare providers and patients to understand the challenges.

**Results**: Unsuccessful TB treatment outcomes (death, loss to follow-up and failure) were higher in new care package (n = 871) compared to conventional care (n = 961) (30.5% vs 23.4%; P value<0.001) and aRR was 1.3(95% CI: 1.1–1.7). Key challenges included patients’ inability to give missed call, increased work load for ART staff, reduced patient–provider interaction, deficiencies in training and lack of role clarity among providers and reduced involvement of TB program staff.

**Conclusion**: With new care package, TB treatment outcomes did not improve as expected and conversely declined compared to conventional care. TB and HIV programs need to address the operational challenges to improve the outcomes.

## Background

India tops the list of high tuberculosis (TB) burden countries accounting for about one-fourth of the estimated global burden [1]. Of the 10.4 million estimated TB patients globally in 2016, 2.8 million were from India. During same period, of the estimated 1 million HIV-infected TB patients globally about 0.1 million (10%) were from India [1,2].

In the past, many countries treated TB patients using an intermittent regimen (drugs consumed twice or thrice weekly) consumed under direct observation of a treatment supporter. Evidence from systematic reviews showed that the relapse and failure rates were 2–3 times higher (with increased risk of acquired rifampicin resistance) among patients receiving intermittent regimen compared to daily regimen, especially among HIV-infected TB patients [3]. To address these concerns, the World Health Organization (WHO) in 2010, recommended daily regimen for drug-sensitive TB patients along with treatment supervision and support to ensure completion of treatment [4,5].

As a method of treatment supervision, ‘Directly observed therapy’ (DOT) has been a subject of controversy and debate [6]. Voices against DOT grew louder following the publication of a systematic review in 2007 which showed that DOT, when compared to self-administered treatment, did not improve treatment outcomes [7]. Also, DOT was viewed as ‘patronising’ and not patient-friendly [8]. While the WHO still recommends DOT, over a period of time, it has come to be viewed as an ‘optional’ patient support measure as opposed to its ‘mandatory’ status before [1].

Traditionally, national TB and HIV programmes have functioned separately with independent staff and distinct service delivery systems, with little co-ordination between them. TB patients had to be referred to HIV clinics and vice versa. This has been changing and the WHO now recommends total integration and ‘one stop service’ for patients with TB and HIV [9].

In this context, a new care package was piloted among HIV-infected TB patients in selected Anti-Retroviral Therapy (ART) centres of India. This pilot had three key components. One, daily treatment regimens using fixed-dose combination drugs was introduced. Two, HIV-infected TB patients received both ART and anti-TB treatment (ATT) at a single-window at the ART centre. Patients were expected to collect drugs once a month and self-administer treatment, given the challenges of organising a daily DOT. Three, an innovative, technology-enabled, adherence support tool called ‘99DOTS’ was introduced [10]. HIV-infected TB patients diagnosed from ART centres not included in the pilot continued to receive conventional care (TB treatment using thrice-weekly intermittent regimens, TB treatment initiation at a health facility nearest to patient’s residence and DOT, ART collected monthly from ART centres).

The pilot created two natural cohorts of HIV-infected TB patients – one receiving the new care package and the other receiving conventional package – and provided an opportunity to assess the effect of the new care package on TB treatment outcomes in programmatic settings. Also, anecdotal evidence indicated several programmatic challenges in shifting from DOT to self-administered treatment using daily regimens. This has not been systematically studied so far. Understanding the implementation challenges will help program managers to take corrective actions and improve the delivery of services. Hence, we conducted a mixed-methods operational research in selected districts of Karnataka State, India to assess (i) the effect of new care package on the TB treatment outcomes compared to conventional care and (ii) the implementation challenges of new care package as perceived by both healthcare providers and HIV-infected TB patients.

## Methods

### Study design

This was an explanatory mixed-methods study with quantitative (cohort study using secondary data collected routinely by the national TB and HIV programmes) and qualitative (descriptive study) components [11].

### Setting

The study was carried out in the state of Karnataka, India with a population of 66.8 million [12]. In 2016, the state had an estimated 0.1 million TB patients with about 11% of them co-infected with HIV [13].The state has about 0.2 million PLHIV with an estimated adult HIV prevalence of 0.45%. The care and support services for PLHIV are provided through 64 ART centres as per national guidelines [14]. Of these, the new care package was implemented in seven ART centres – one each in the districts of Bangalore city, Belgaum, Gulbarga, Mysore, Davanagere and two centres in Bagalkot district.

### Diagnosis of TB among PLHIV

All PLHIV are routinely screened for TB using ‘four symptom complex’ (fever, cough, weight loss and night sweats) during each of their visit to the ART centre. In people with symptom(s), sputum specimens are collected and sent for sputum microscopy and Xpert MTB/RIF® assay for TB diagnosis. If Xpert® facility is not co-located within the ART centre, then the sample is sent to the nearest Xpert® facility for testing. TB is diagnosed and treated as per national guidelines which are in line with WHO guidelines [15,16].

### New care package

The new care package had three key components (Table 1) and is briefly described below.

**Table 1.** Comparison of components of new and conventional care for HIV-infected TB patients in selected districts of Karnataka state, India between January and December 2016.

Components	New care package	Conventional care
Screening for TB	Four symptom-based intensified screening at ART centre	Four symptom-based intensified screening at ART centre
Diagnosis of TB	Using CBNAAT and sputum microscopy facilitated by ART staff	Using CBNAAT and sputum microscopy facilitated by ART staff; Sputum samples were collected and transported to the nearest CBNAAT facility, wherever applicable
TB Treatment Initiation	Immediately after TB diagnosis at the ART centre	Patient referred from ART centre after diagnosis for treatment initiation at a Peripheral Health Institution (PHI), near to patient’s residence
Type of Drugs	Fixed-dose combination (FDC) of 4FDC during intensive phase and 3FDC during continuation phase. The number of pills per patient per day ranges from 2 to 5 based on weight band	No FDC available. Individual drugs with fixed-dose for all adults. The number of pills per patient (thrice weekly) ranged from 7 during intensive phase to 5 during continuation phase
Dosing frequency	Daily	Thrice weekly (alternate days)
Adherence support and compliance monitoring	Self-administered treatment with 99DOTS (Technology-enabled adherence support)	Directly Observed Treatment, usually provided by community health worker/volunteer of general health system
Drug dispensing	Dispensed for 28 days from the ART centre during their routine scheduled visit to centre	Dispensed thrice weekly during intensive phase and once weekly during continuation phase of treatment by the DOT provider at a place convenient to the patient
Patient Follow-up	ART centre	Peripheral Health Institution
Treatment outcome ascertainment	By the ART medical Officer	By the Medical officer of PHI

*Abbreviations*: HIV- Human Immunodeficiency Virus; TB- Tuberculosis; ART- Antiretroviral Therapy; CBNAAT- Cartridge-Based Nuclei Acid Amplification Test; FDC- Fixed-Dose Combination; PHI- Peripheral Health Institution; DOT- Directly Observation Treatment

*Daily Regimen*: The newly diagnosed TB patients receive two months of intensive phase (2HRZE: H = Isoniazid, R = Rifampicin, P = Pyrazinamide, E = Ethambutol) and four months of continuation phase (4HRE) while previously treated patients receive three months of intensive phase (2HRZES + 1HRZE; S = Streptomycin) and five months of continuation phase (5HRE). Fixed-dose combination (FDC) drugs of HRZE (4FDC) and HRE (3FDC) are provided. The number of FDC tablets to be consumed per day ranges from two to five based on weight band of the patient.

*Single window system*: Once the PLHIV is diagnosed with TB, the ART is started at the ART centre. The ART nurse or the counsellor provides counselling regarding adherence, usage of 99DOTS, and possible side effects of anti-tubercular treatment (ATT). The nurse prepares two copies of the TB treatment card. One copy is kept at the ART centre and is updated once per month after the pill count during patient visit. The other copy is given to the patient and is expected to be updated by the National Tuberculosis Programme (NTP) field staff during their regular house visit. Under single window system, patients are given both ATT and ART medication at the ART centres during their monthly visits.

Every week, the ART nurse shares the list of the newly diagnosed TB patient with DOTS Plus Supervisor (DPS) of the NTP. The DPS in turn informs the Senior Treatment Supervisor (STS) of the respective tuberculosis units (TU, a sub-district level administrative unit for every 250,000 population). After making the initial house visit, the STS registers the patient in the TB treatment register and is expected to link the patient to a ‘treatment supporter’ (community health worker/volunteer) to facilitate the treatment adherence and follow-up.

*99DOTS*: It is an innovative mHealth tool that uses basic mobile phones to monitor and improve adherence to TB drugs. After consuming the tablets, the patient is expected to give a missed call from the registered mobile phone number to the toll-free number printed on the envelope of the blister pack [17]. In case the patient fails to give a call, an alert message is sent to STS and to the treatment supporter facilitating the treatment. In case of a missed dose, the treatment supporter is expected to visit the patient and carry out the retrieval action. The STS is expected to update the details of retrieval action and reasons for the missed doses in the 99DOTS web portal.

The mode of delivery of the three components of new care package is explained in detail in the guidelines released by the National AIDS control programme prior to universal implementation of new care package in 2017 [18].

### Conventional care

Once the individual was diagnosed with TB, the ART medical officer refers the patient to the peripheral health institution (PHI) nearest to the patient’s residence for initiation of TB treatment. The drugs and duration of treatment remain the same as above, except the dosing frequency is thrice weekly instead of daily, under the direct supervision of the DOT provider. Contact details, morbidity details and medication adherence, follow-up details and TB treatment outcomes of these patients are documented in individual TB treatment card maintained by the DOT provider. Patient continues to receive ART at the ART centres and the ART drugs are collected by the patient once a month.

### Data recording and reporting

STS documents the details of HIV-infected TB patients residing in his/her TU in a MS Excel database which include type of TB, regimen, adherence support, date of initiation, treatment outcomes, date of treatment outcome, CD4 count and ART status. The DPS collates all the TU-level details into a master database at the district level and updates monthly. This electronic database is shared between NTP and HIV program staff.

### Study site

**Figure 1.**
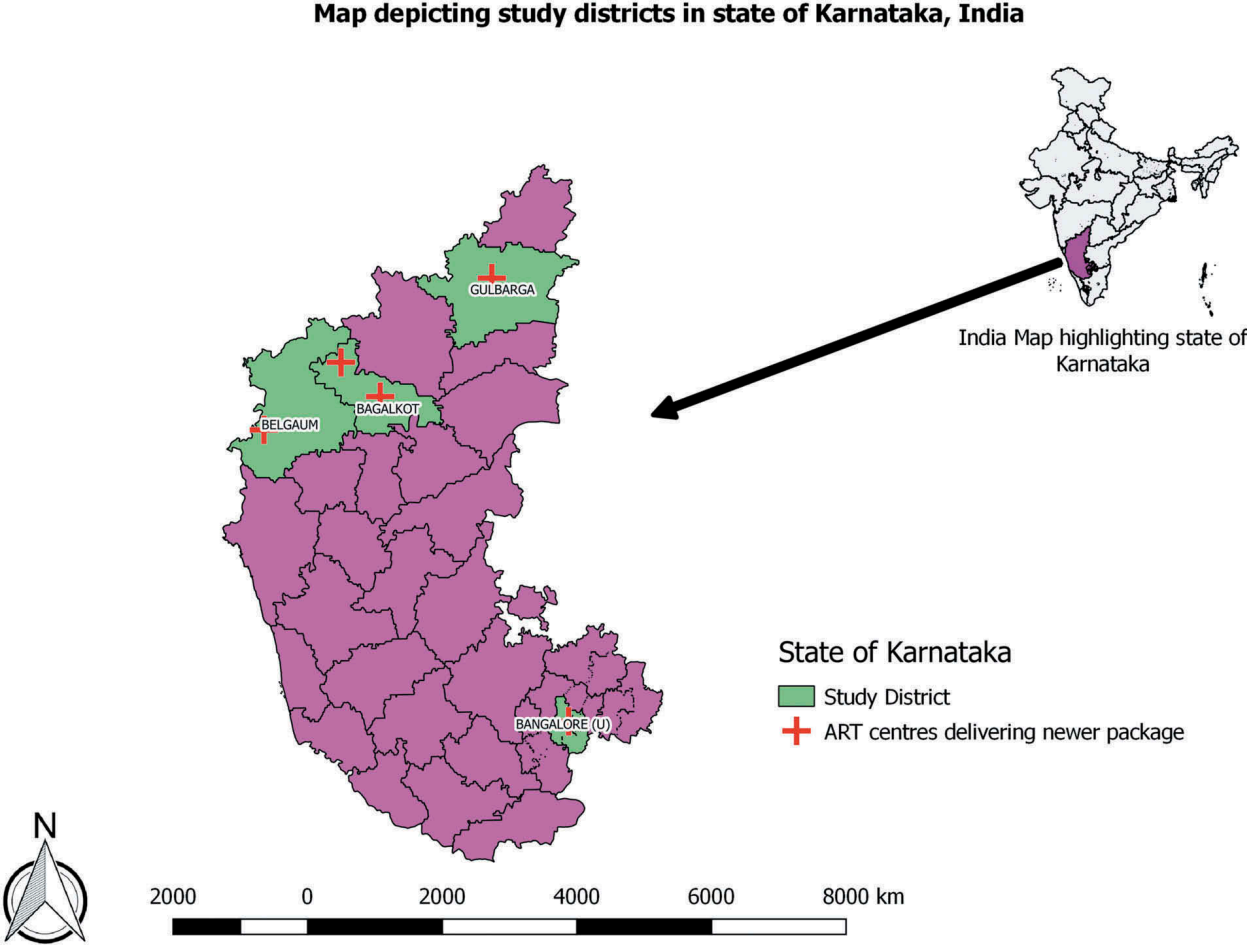
Map of study districts and ART centres delivering new care package in the state of Karnataka during January to December, 2016.

The study was conducted in 21 ART centres situated in four districts of Karnataka State (Belgaum, Gulbarga, Bagalkot and Bangalore city) (Figure 1). These districts were purposively selected considering that at least one ART centre in the district had provided new care package during pilot phase and represented the four different administrative regions of the state of Karnataka. Five out of the 21 ART centres in these districts delivered new care package.

### Study population

#### Quantitative

The study included all HIV-infected TB patients registered between January and December 2016. Patients with drug resistant TB were excluded.

#### Qualitative

Study population included HIV-infected TB patients (n = 3) and healthcare providers involved in delivery of new care package (n = 17). We used a purposive sampling to select health care providers with representation of different cadres of staff of both TB and HIV programmes. This included two District Tuberculosis Officers, three DPS, two ART Medical Officers, three ART counsellors, four STS and three Tuberculosis Health Visitors. The HIV-infected TB patients who received the new care package were conveniently selected and included.

### Data collection

#### Quantitative

Data on age, gender, type of TB (Microbiologically confirmed TB/Clinically diagnosed), site of TB, treatment category, type of ATT (daily/intermittent), ART status, latest available CD4 cell count, and TB treatment outcome was extracted from the electronic TB/HIV-line list. The TB treatment outcome definitions are as recommended by the WHO and are listed in **Supplement Table-1**.

#### Qualitative

In-depth interviews were conducted face-to-face in local language (Kannada) by PT (a male medical doctor, MD, trained in qualitative research) and audio recorded using ‘Voice Recorder’ mobile app after obtaining consent. Only one participant did not consent for audio-recording, when notes were taken. The interviewer is working in an international non-governmental organisation and was not involved in program implementation. Separate interview schedules with probes were used to interview patients and health care providers. The interviews of health care providers were conducted at their work place. Patients were interviewed at their place of convenience (two at ART centre and one at patient’s workplace). Interviews lasted for an average of 26 minutes (range 13–47 minutes). At the end of each interview, debriefing was done to ensure member checking.

### Data analysis

#### Quantitative

Data from the four districts were analysed using Stata 12.0. Descriptive statistics were computed on demographic, clinical and treatment characteristics, and TB treatment outcomes. Patient characteristics were compared across the two groups using either Chi-square test or Mann Whitney U test.

We combined ‘death’, ‘loss to follow-up’ (LFU), ‘failure’ and ‘transfer out’ as ‘unsuccessful’ outcomes (as most of the LFU cases might be unaccounted deaths among HIV-infected TB patients and failure and transfer out proportions were low). Similarly ‘cured’ and ‘treatment completed’ were considered ‘successful outcome’. Treatment outcomes between patients on new care package and conventional care were compared using Chi-square test.

To assess the independent effect of the new care package on ‘Unsuccessful’ TB treatment outcome, a cluster adjusted generalised linear model (Poisson model) was used to adjust for confounding variables (age, sex, type of TB, site, history of TB treatment, latest CD4 cell count, ART status) and the adjusted relative risks with 95% confidence intervals was calculated [19,20].

#### Qualitative

Transcripts were prepared within a week of conducting the interviews by PT. Descriptive content analysis was performed manually by two independent, trained researchers (KS and VV) to generate categories and themes. These were reviewed by the PT and AMVK to mitigate subjectivity in analysis. The results were reported using ‘Consolidated Criteria for Reporting Qualitative Research’ [21].

## Results

The study included 870 patients who received new care package and 961 patients who received the conventional care package. Age and gender distribution was similar between the two groups (Table 2). The two groups varied by geographical location (districts) and clinical profile such as microbiological confirmation, site of TB, ART status and CD4 counts.

**Table 2.** Comparison of socio-demographic and clinical characteristics of HIV-infected TB patients registered for TB treatment with either new care package or conventional care package in selected districts of Karnataka between January 2016 and December 2016.

Characteristics	New care package, n (%)*	Conventional care, n (%)*	p Value^†^
**Total**	**870**	**961**	
**Age in years**			
0–14	13 (1.5)	33 (3.4)	0.041
15–29	110 (12.6)	135 (14.1)	
30–44	447 (51.4)	482 (50.2)	
45 and above	300 (34.5)	311 (32.4)	
**Gender**			
Male	546 (62.8)	597 (62.1)	0.779
Female	324 (37.2)	364 (37.9)	
**District**			
Belgaum	226 (26.0)	558 (58.1)	< 0.001
Gulbarga	185 (21.7)	47 (4.9)	
Bagalkot	425 (48.9)	144 (15.0)	
Bangalore	34 (3.9)	212 (22.1)	
**Type of TB**			
Microbiologically confirmed	283 (32.5)	507 (52.8)	<0.001
Clinically diagnosed	587 (67.5)	454 (47.2)	
**Site of TB**			
Pulmonary TB	732 (84.1)	698 (79.5)	0.010
Extra Pulmonary TB	138 (15.9)	156 (20.5)	
**Category of TB Treatment**			
New	708 (81.4)	789 (82.1)	0.689
Previously treated	162 (18.6)	172 (17.9)	
**ART status**			
On ART	782 (89.9)	829 (86.3)	0.017
Not on ART	88 (10.1)	61 (13.7)	
**CD4 count (cells/mm3)**			
Less than 50	91 (10.5)	66 (6.9)	<0.001
50–199	384 (44.1)	353 (36.7)	
200–349	168 (19.3)	215 (22.4)	
350–499	83 (9.5)	94 (9.8)	
More than 500	61 (7.0)	81 (8.4)	
Not available	83 (9.5)	152 (15.8)	
**CD4 count (Median (IQR))**	160 (83–280)	194 (100–315)	0.001^#^

*Column Percentage, ^†^Chi-square test, ^#^Mann Whitney U test

*Abbreviations*: HIV- Human Immunodeficiency Virus; TB- Tuberculosis; ART- Antiretroviral Therapy; CD4- Cluster of Differentiation 4/CD4 + T helper cells

TB treatment outcomes are summarised in Table 3. Overall, 73.2% of patients had successful outcomes. Among patients receiving new care package, 30.5% (95% CI: 27.4–33.6) had unsuccessful outcomes compared to 23.4% (95% CI: 20.8–26.2) on conventional care (P value<0.001). We did a post-hoc power calculation based on the principles of comparison of two sample proportions using Pearson Chi-square test with alpha error of 5%. Estimated power for difference in unsuccessful outcome was 93%. Both death (20%) and lost to follow up (9.1%) were higher among patients in new care package compared to conventional care, but proportion cured was lower (10.9% vs 30.5%).

**Table 3.** Comparison of TB treatment outcomes among HIV-infected TB patients registered for TB treatment with either new or conventional care package in selected districts of Karnataka from January to December 2016.

TB treatment Outcome	New care package, N = 870, n (%)*	Conventional care package, N = 961, n (%)*	p Value^#^
**Successful**	**605 (69.5)**	**736 (76.6)**	**<0.001**
Cured	95 (10.9)	293 (30.5)	<0.001
Treatment Completed	510 (58.6)	443 (46.1)	<0.001
**Unsuccessful**	**265 (30.5)**	**225 (23.4)**	**<0.001**
Failure	2 (0.2)	0 (0.0)	-
Lost to follow up	79 (9.1)	55 (5.7)	0.005
Died	174 (20.0)	152 (15.8)	0.019
Shift to Cat IV	1 (0.1)	6 (0.6)	0.083
Transfer Out	9 (1.0)	12 (1.3)	0.549

^*^Percentage calculated with total number of individuals in each group as denominator

^#^Chi-square test

*Abbreviations*: HIV- Human Immunodeficiency Virus; TB- Tuberculosis; Cat IV- Drug regimen used to treat Multi Drug Resistant TB patients

In adjusted analysis, new care package was independently associated with unsuccessful outcomes. Patients receiving new care package had 30% higher risk of unsuccessful outcomes compared to conventional care (adjusted RR 1.3; 95% CI: 1.1–1.7) as shown in Table 4. In the cluster adjusted model, the intraclass correlation (ICC) coefficient at Tuberculosis Unit (TU) level was found to be 0.01 (95% CI: 0.001–0.059) indicating no significant clustering.

**Table 4.** Association of socio-demographic, clinical and treatment-related factors with unsuccessful outcomes among HIV-infected TB patients registered for TB treatment in selected districts of Karnataka from January to December 2016.

Characteristic	Total	Unsuccessful outcome, n (%)*	Unadjusted RR (95% CI)	Adjusted RR (95% CI)^#^	p value
**Total**	**1831**	**490 (26.7)**			
**Age (in years)**					
0–14	46	9 (19.6)	1	1	
15–29	245	57 (23.3)	1.2 (0.6–2.2)	1.2 (0.7–2.1)	0.501
30–44	929	246 (26.5)	1.4 (0.7–2.5)	1.3 (0.7–2.3)	0.354
45 and above	611	178 (29.1)	1.5 (0.8–2.7)	1.4 (0.8–2.3)	0.254
**Gender**					
Male	1143	329 (28.8)	1.2 (1.0–1.4)	1.1 (0.9–1.4)	0.171
Female	688	143 (23.4)	1	1	
**Type of TB**					
Microbiologically confirmed	790	215 (27.2)	1.0 (0.9–1.2)	1.1 (0.8–1.4)	0.562
Clinically diagnosed	1041	275 (26.4)	1	1	
**Site of TB**					
Pulmonary TB	1496	401 (26.8)	1	1	
Extra Pulmonary TB	335	89 (26.6)	1.0 (0.8–1.2)	1.1 (0.9–1.3)	0.336
**Category of Treatment**					
New	1497	395 (26.4)	1	1	
Previously treated	334	95 (28.4)	1.1 (0.9–1.3)	1.1 (0.9–1.5)	0.330
**District**					
Bangalore	246	48 (19.5)	1	1	
Belgaum	784	218 (27.8)	1.4 (1.1–1.9)	1.4 (0.9–2.1)	0.094
Bagalkot	569	154 (27.1)	1.4 (1.0–1.8)	1.1 (0.7–1.7)	0.743
Gulbarga	232	70 (30.2)	1.5 (1.1–2.1)	1.4 (0.9–2.2)	0.142
**ART status**					
On ART	1611	358 (23.7)	1	1	
Not on ART	220	109 (49.6)	2.1 (1.8–2.5)	2.1 (1.6–2.9)	<0.001
**CD4 count (cells/mm3)**					
Less than 50	157	59 (37.6)	2.1 (1.4–3.2)	2.0 (1.4–2.9)	<0.001
50–199	737	209 (28.4)	1.6 (1.1–2.3)	1.6 (1.1–2.3)	0.010
200–350	383	81 (21.2)	1.2 (0.8–1.8)	1.2 (0.8–1.8)	0.292
350–500	177	33 (18.6)	1.1 (0.7–1.7)	1.1 (0.6–1.8)	0.756
More than 500	142	25 (17.6)	1	1	
Not available	235	83 (35.3)	2.0 (1.4–3.0)	1.6 (1.0–2.5)	0.062
**TB treatment regimen**					
New care package	870	265 (30.5)	1.3 (1.1–1.5)	1.3 (1.1–1.7)	0.014
Conventional care package	961	225 (23.4)	1	1	

*Row percentage

^#^Model adjusted for clustering at 38 Tuberculosis Units included in the study

*Abbreviations*: HIV- Human Immunodeficiency Virus; TB- Tuberculosis; RR- Relative Risk; CI- Confidence Interval; ART- Antiretroviral Therapy; CD4- Cluster of Differentiation 4/CD4 + T helper cells

### Challenges in implementation of the new care package

**Figure 2.**
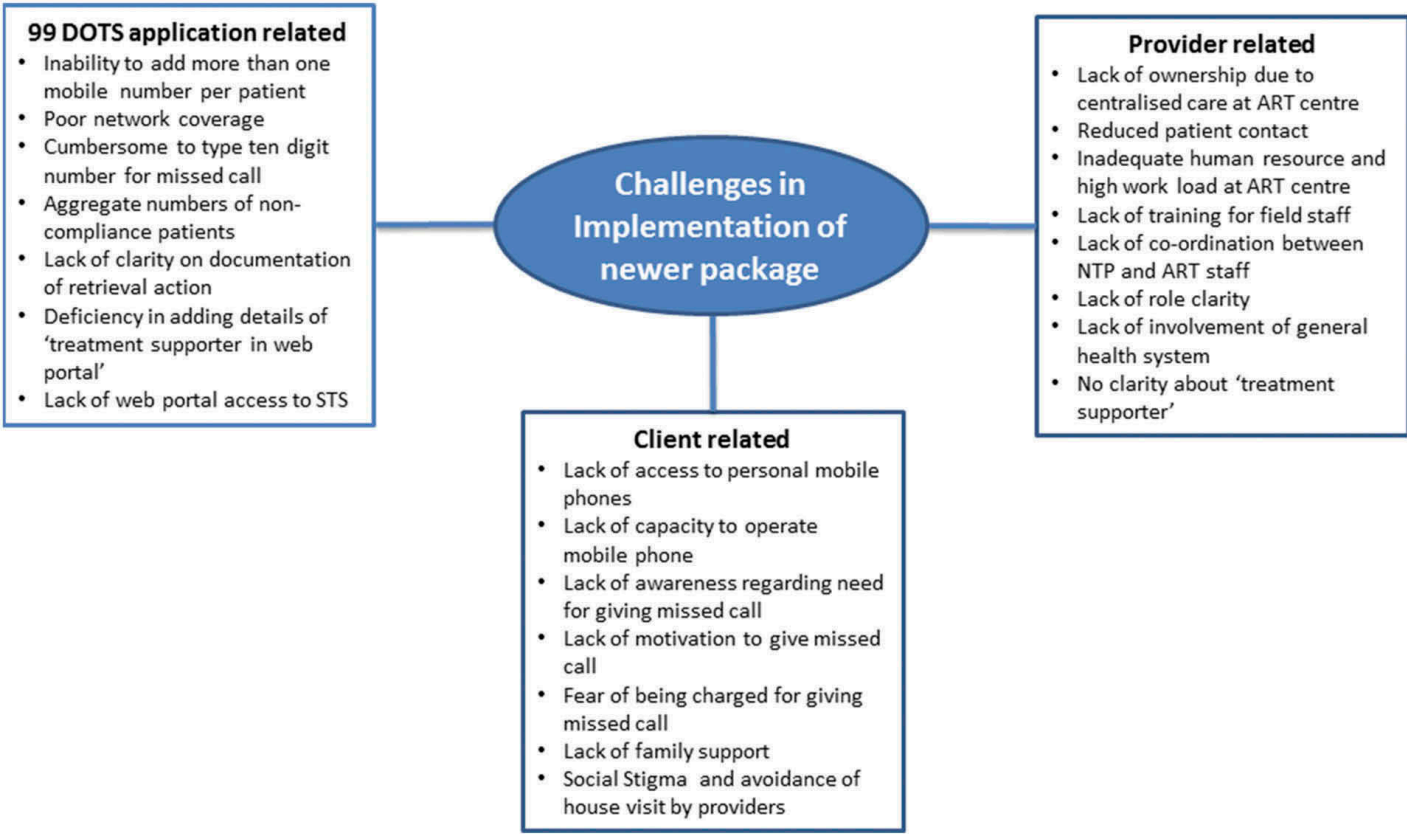
Challenges in implementation of new care package among HIV-infected TB patients in selected districts of Karnataka from January to December 2016.

In total, 21 codes related to challenges were deduced from the transcripts and were categorised into three main categories. This has been summarised in Figure 2 and briefly described below.

### Patient-related challenges

Healthcare providers noted that patients faced challenges with 99DOTS, failing to give missed phone call. Some patients do not own mobile phones and have to rely on family members, who may not always be present or supportive. Some patients do not know how to use phones. Lack of motivation for giving missed call’ and ‘lack of awareness on need for giving missed call’ were other challenges.

One of the ART staff opined

‘Majority of the times, uneducated illiterate people will be there, villagers…, elderly grandmother and grandfathers will be there. I tell them, “you need to take these one, two and three tablets, then there will be a number and you need to call”. They will tell “how will I know all these, we don’t know how to operate the mobile phone”. Or few will say “we don’t have anyone, we don’t have any mobile, I stay alone or only I and my wife stay together”. In such situations we don’t know what to do.’

### 99DOTS application-related challenges

Health care providers and patients perceived that there were few deficiencies in the 99DOTS application like; inability to register more than one phone number per patient, difficulty for patients to dial 10 digit number and absence of automated voice reply. Patients expected that someone will talk to them on giving call to 99DOTS portal and this led to confusion.

‘Few had difficulty in using those 10 digit number to call. Also when they come here, they would have given some relatives phone number. But the relative may not be there every time with the patient.’

A patient noted,

‘*Around seven or eight days as soon as I used to take tablets, I used to try at least three or four times. Few days I even tried ten times. But it never connected sir, never connected. I used to try from morning up to 10:30 am. But it never connected, what we can do sir (laughs).’*

Given these challenges, the healthcare providers started believing that ‘not getting missed call’ is because of these challenges rather than ‘not consuming drugs’. This meant that no action was taken on not receiving the missed call.

*‘Not more than 50%* [give missed call]. *But they will take tablet.’- HCP*

Healthcare providers highlighted issues with alert messages sent from 99DOTS portal when patients were not giving missed call. The alert messages contained feedback in aggregate numbers rather than on details on individual patients who did not consume drugs. Healthcare provider had to visit the web portal to know about individual patients and accessing the portal was difficult in field conditions. Also, they complained that there was no provision to document the retrieval action after missed dose.

‘Before the intimation was specific about particular patient did not take the drugs. But now the message is like out of 30 people in your area 22 are taking tablets regularly and remaining did not take properly’

### Provider-related challenges

Healthcare providers felt that the centralised TB treatment at ART centre without involving general health system or TB program staff had reduced the number of the patient–provider interactions and leading to an increase in the ‘loss to follow-up’.

‘Here there was no contact between the RNTCP staff and clients. Everything is delivered in ART centres. We don’t have any field workers. Our interaction will be only when they come once in month to ART centre. We come to know about his medication only after one month. This one month is a big gap.’

With the single window system of drug dispensing, ART centre staff felt that their workload had increased and the involvement of the NTP staff was minimal. This was acknowledged by the NTP staff too.

‘We feel very bad about it. ART drug delivery itself is a big load, we need to give drugs, counsel and also follow-up. Along with that adding this has become a burden for us… Also there is manpower problem. Because of shortage of manpower, we cannot have a single person constantly looking after TB/HIV patients’

One of the NTP staffs said,

‘In regular DOTS we will only visit home, counsel them. So they will come to know that we are only providing all care when we do first visit. But in 99DOTS, drug provider is different, we are different, and person following up weekly is different.’

NTP staff noted that they were not clear about their roles in the new care package and also about who can be ‘treatment supporter’.

‘We are not aware of the “treatment supporter”. We did not get any training regarding it’

One of the common challenges noted by healthcare providers was the lack of training on the new care package for the NTP and ART staff. They also felt that only few cadres of staff were trained and there was no joint training for NTP and ART staff which could have clarified their roles.

‘There were few trainings. But there was no joint training for both RNTCP and ART staff. But what these people do is, they call from ART centre only ART-MO and ART staff nurse… In RNTCP, they call DTO and DPS,… STS or TB-HV will be completely unaware of it.’

### Positive aspects of the new care package

Both healthcare providers and patients consistently voiced that the patients’ complaints on pill burden and the adverse drug effects decreased with the introduction of the FDC drugs. One PLHIV who had received both conventional and new care package, when asked about the regimens said,

‘Sir, taking two tablets (99DOTS) is better sir. There was no problem at all sir. No fever, no tiredness nothing was there. I can eat food properly, I can work freely, I work in home also. There is no problem.’

## Discussion

This is the first study assessing the effect of the new care package of daily regimen, single window system and 99DOTS system on TB treatment outcomes of HIV-infected TB patients. The study showed a 30% higher chance of unsuccessful TB treatment outcomes among patients who received the new care package compared to patients receiving conventional care. Client’s inability to give missed phone call, increased work load for ART staff, reduced patient-provider interaction, the lack of joint staff training prior to the implementation, lack clarity about their role among the healthcare providers and less involvement of NTP staff were the important challenges identified.

In this study, TB treatment outcomes did not improve as expected and conversely declined compared to conventional care. Being the first study assessing the effect of this novel new care package, we do not have any studies to compare our findings. However, evidence is available to support that daily regimen improves TB treatment outcomes. A recent randomised controlled trial from India among HIV-infected TB patients confirmed the beneficial effect of daily regimen [22]. Hence, it is unlikely that daily dosing contributed to the poor outcome.

The new care package employed self-administered treatment with technology-enabled adherence support over conventional DOT strategy. The recent systematic review on different strategies for TB treatment support reported that DOT through trained providers would improve adherence and treatment outcomes over unsupervised self-administered treatment [23]. In new care package, self-administered treatment was expected to be supported and monitored through 99DOTS. However, poor implementation of 99DOTS meant that the treatment was mostly unsupervised. Also, due to the single window system, the patient-provider interaction happened only once in 30 days, whereas in conventional care they met frequently, on alternate days or once a week. Limited patient-provider interaction meant lesser opportunities for treatment adherence counselling and motivation. Thus, the challenges in the effective implementation of 99DOTS and the single window system might be the reasons for the poor performance of the new care package.

Qualitative study done with the stake holders identified several challenges related to 99DOTS. Patient’s inability to give missed call for various reasons led to poor monitoring of adherence to TB treatment. Educating the patients on the meaning and importance of missed call, improving the system to allow registration of more mobile phone numbers and a better patient support system (DOT) for those without access to mobile phones, is the way forward. Even though 99DOTS had the potential to reduce the number of health facility visits, the poor implementation of the programme associated with the complete absence of DOT might have led to higher LFU rates in the new care package cohort. As TB treatment was started at ART centre itself, neither NTP staff nor the community healthcare workers made house visits or follow-up on these patients. This limited the retrieval action of poorly adherent patients. Even with the option of adding a community health worker/volunteer as a ‘treatment supporter’ existed in the package, poor definition of roles and responsibilities coupled with lack of training led to poor implementation. There was no ownership of patients by the NTP staff.

Adding a local ‘treatment supporter’ might help in frequent interaction with the patients, providing a quicker response to the alert message of missing a dose, tracking of the LFU patients and linking the patients with general health system in case of adverse effect. Also providing a joint training of the NTP field staff and ART staff will be helpful in clarifying their roles better and make them accountable. Combined review meetings of TB and HIV program staff should include indicators related to new care package like completeness of retrieval action, house visits and random pill counts during house visits.

The study has several strengths. First, we used a mixed methods design. The qualitative component brought out the important challenges related to the low performance of the new care package. Second, we used routinely collected programmatic data for the quantitative component and thus difference in the treatment outcomes reflects the realities in the field. Third, we had a large sample size powered enough to detect the difference between the two packages. Fourth, we adhered to the Strengthening the Reporting of OBservational Studies in Epidemiology (STROBE) guidelines and to the COREQ guidelines for reporting the quantitative and qualitative components, respectively.

The study has some limitations. We could not tease out the effect of individual components of new care package on treatment outcomes. We relied on the routinely collected program data and hence recording errors cannot be ruled out. However, missing information was very less except for CD4 count. There were differences in the baseline characteristics between the two groups. While we performed adjusted analysis, residual confounding due to other factors like delay in initiation of treatment, nutritional status, and educational status cannot be ignored. We did not have information on process indicators like number of patients who gave missed call or the correctness of mobile numbers registered. Finally, for understanding the challenges from client’s perspective, we could interview only three patients and might not have achieved saturation. Therefore, the patient-related results should be interpreted with caution. However, it was reassuring to find that the interviews among patients reflected similar implementation challenges as those shared by the healthcare providers.

## Conclusion

This study revealed no improvement in the TB treatment outcomes when using the new care package for TB treatment of HIV-infected TB patients. There is a need for addressing the programme implementation challenges like lack of training, patient side issues in giving missed call and low involvement staff of NTP and general health system. Effect of package on treatment outcomes needs to be re-assessed after addressing the above challenges.

## Paper context

This is the first study assessing the TB treatment outcomes and implementation challenges of new care package (self-administered daily regimen with fixed-dose combination, ‘one stop service’ at Antiretroviral Treatment (ART) centre and 99DOTS) for treatment of HIV-infected TB patients. The new care package had 30 percent higher chance of having unsuccessful treatment outcomes compared to conventional care. The implementation challenges need to be addressed before scaling up this strategy.
