# Supplementary material for: Outcomes and implementation challenges of using daily treatment regimens with an innovative adherence support tool among HIV-infected tuberculosis patients in Karnataka, India: a mixed-methods study
Source: Glob Health Action. 2019 Feb 4;12(1):1568826. doi: 10.1080/16549716.2019.1568826 (PMC6366428; doi:10.1080/16549716.2019.1568826)
Supplement: Supplemental Material [file ZGHA_A_1568826_SM4747.docx]

**Supplement Table 1**: Definitions of treatment Outcomes of HIV-infected TB patients in Karnataka State, India

| **Programmatic Outcomes** | **Definition** |
| --- | --- |
| **Cured** | A TB patient who was microbiologically confirmed for TB at the beginning of treatment but who is smear or culture negative at the end of complete treatment |
| **Treatment completed** | A TB patient who completed treatment without evidence of failure or clinical deterioration BUT with no record to show that the smear or culture results of biological specimen in the last month of treatment was negative, either because the test was not done or because the result is unavailable |
| **Treatment success** | TB patients either cured or treatment completed are accounted in treatment success |
| **Failure** | A TB patient whose biological specimen is positive by smear or culture at the end of treatment  Failure to Respond: A child of paediatric TB who fails to have bacteriological conversion to negative status or fails to respond clinically/or deteriorates after 12 weeks of completion of intensive phase shall be deemed to have failed response, provided alternative diagnoses/reasons for non-response have been ruled out |
| **Lost to follow up (LFU)** | A TB patient whose treatment was interrupted for one consecutive month or more |
| **Not evaluated** | A TB patient for whom no treatment outcome is assigned; this includes former ‘transfer-out’ patients |
| **Treatment regimen changed** | A TB patient who is on first line regimen and has been diagnosed as having DR-TB and switched to drug resistant TB regimen prior to being declared as failed |
| **Died** | A patient who has died during the course of anti-TB treatment |

*Abbreviation:* HIV- Human Immunodeficiency Virus; TB- Tuberculosis; DR-TB- Drug Resistant Tuberculosis
